# The Cross Contamination (Cross Colonization) Phenomenon of Probiotic Use in Neonatal Intensive Care Units: Putative Mechanisms and Clinical and Research Implications

**DOI:** 10.7759/cureus.2691

**Published:** 2018-05-25

**Authors:** Karthikeyan Gengaimuthu

**Affiliations:** 1 Neonatology and Pediatrics, International Modern Hospital, Dubai, ARE

**Keywords:** cross colonization, probiotics, neonates, hand hygiene

## Abstract

In studies of probiotic use in neonates the phenomenon of cross contamination (cross colonization) of the control group neonates with the probiotics administered to the study group was observed and a hypothetical reanalysis of the presented data after statistically controlling this phenomenon unveils significant benefits resulting from probiotic therapy. This article discusses the putative pathogenesis of this phenomenon and its clinical and research implications.

## Introduction

Lactobacillus species, bifidobacterium species, streptococcus thermophilus and saccharomyces boulardii are the probiotic strains that have been used in neonates [1]. Meta-analysis of studies on probiotic use in neonates done outside western centers had shown significant benefits of probiotics in reducing the incidence of necrotizing enterocolitis and all-cause mortality [2]. In the recent well-conducted randomized placebo-controlled trials (Probiotics in Preterm Infants Study (PiPS) from the UK and ProPrems study from Australia) on the probiotic use in neonates, it was observed that colonization of gut with the probiotics that confers the intended putative benefits of probiotics is not restricted to the study group who received the probiotics but also occurs to a significant extent in the control group neonates as well [3,4]. The PiPS study reports in the article itself that this happens to an extent of 49% in the control group [3]. The ProPrems study had acknowledged the existence of this phenomenon in the discussion part of the article [4] and this is reported in detail in a subsequent publication from the same study group [5]. This cross ‘contamination’ phenomenon benefits the control group neonates to a level that confounds the published results and declared conclusions. In a hypothetical reanalysis that eliminates this significant confounding factor of cross-contamination phenomenon in the PiPS trial from the UK, it was demonstrated that Bifidobacterium brevi, the probiotic used in that trial confers significant benefits in reducing all the three outcome measures necrotizing enterocolitis, late-onset sepsis and all-cause mortality [6]. In this article, we will explore the putative mechanisms of this cross-colonization (cross contamination) phenomenon and its clinical and research implications.

## Technical report

Mechanism of cross-contamination of probiotics in neonatal intensive care units (NICU)

The bacterial probiotics used in the studies in neonates are gram-positive viable organisms and are prepared from lyophilized powder form in sachets. Hand washing and alcohol-based gels are the mainstays of prevention of cross-infection in the current neonatal units. It has not been mentioned in the UK and Australian probiotic studies whether gloves were uniformly used during preparation of probiotic dosage for administration.

Lyophilized sachet reconstitution process can result in a significant environmental contamination. Although we could not find any study that explores this in the context of bacterial probiotics used in neonates, there is one published study that has investigated this phenomenon with the non-bacterial probiotic Saccharomyces boulardii. The mere opening of sachets of lyophilized saccharomyces boulardii leads to significant environmental contamination (probiotic blast) that included air at 1-meter distance, simulated patient surface and hands of the sachet opening operator, the last being the heaviest that persists even after hand washing. It was recommended in this article that preparation of saccharomyces boulardii for clinical use should be done outside the patient care area using gloved hands [7]. It is plausible that the probiotic bacteria persist on the health care worker’s hands till the time of handling control group neonate (the timing of this cannot be known retrospectively) and routine hand care practices during the study period fail to prevent the feco-oral route of probiotic contamination (transmission) to the control group neonates. Currently recommended hand hygiene measures for infection control in intensive care units like handwashing, handrubs with alcohol and chlorhexidine and even gloving do not offer flawless protection against contact transmission [8, 9]. It is known that only the transient but not the resident skin flora are completely removed by alcohol hand rubs, the latter requiring surgical hand scrub routine [8]. It is possible that probiotic bacteria share the same inherent characteristics of resident skin flora.

The next question is whether a single sentinel event of feco-oral transmission is enough for ‘contamination’ of the control group. The answer to this question is probably negative as it has been known that gram-positive probiotic bacteria are only transient colonizers of the gut [10]. Hence the gram-positive probiotics lactobacillus and bifidobacterium species have to be administrated everyday to the study group to maintain colonization and the effect wanes away (meaning the feces test negative for the probiotic bacteria) after stopping regular probiotics [11]. Logical extension of this analogy means that contamination of the control group is also not due to a one-off event but was also equally regular as colonization of stools with probiotic takes 5–7 days of regular administration [11]. It is mentioned in the methodology section of PiPS study that the preparation of the dosage of probiotic was done in the milk kitchen of the units and hence this contamination is not only due to the contamination of the hands of the sachet operator during the process of preparation but should include additional mechanisms like transmission from the feces of the colonized study group neonates. Gram-positive bacterial cell walls are thicker than gram-negative bacterial ones and hence alcohol hand rubs are more effective in removing gram-negative bacteria from the hands, the mechanism being the dissolution of lipids in the cell wall and is the core concept of the grams stain preparation [12]. Simulation experiments are needed to understand the usefulness of current hand hygiene strategies in preventing cross-transmission of probiotic bacteria by healthcare workers in NICUs but it has to be noted that bifidobacterium species bacteria are notoriously difficult to grow in culture media [11]. If the feco-oral route is not the causative one then the only possibility remains is that there was constant contamination of the milk meant for the study group babies from the milk room where the sachets of lyophilized probiotic were opened.

However, this phenomenon of cross contamination occurred to a lesser extent in the Australian study as well wherein the preparation of the probiotic was by the hospital pharmacist and the entire clinical team was blinded [5]. A critical appraisal of this published article throws interesting light on this cross-contamination phenomenon (cross-colonization as it has been termed in this article from Australia).

1. Stool and environmental samples were collected for molecular DNA fingerprinting evidence for probiotic bacteria colonization (presence of any two of the three probiotic bacteria used in the ProPrems trial, Bifidobacterium lactis, Bifidobacterium infantis and Streptococcus thermophilus being termed as positive) at two predefined time points, one during the study period (Point A) and second six months after the first time point during off the study period (Point B).

2. Forty-three stool samples (five from probiotic group, seven from placebo group and 31 from non-study group infants) and 19 environmental samples (12 from rooms where probiotic-administered infants were there and seven from other rooms) were obtained during point A.

3. Forty-four stool samples and eight environmental samples were obtained during point B.

4. At point A all five of five probiotic group stool samples were positive, two of seven in the placebo group and one of 31 in the non-study group were also colonized thus giving the cross-colonization rate of three of 38 (7.9%). This is well and way below the stool colonization frequency of placebo group in the PiPS study (around 40%). This implies that the more distant the probiotic preparation site (non-involvement of nursery personnel) from the infants lesser the chance of cross-colonization.

5. At point A, four of 12 environmental samples from rooms wherein probiotic administered infants were cohorted were positive which effectually means that the probiotic bacteria in the environment are indeed sourced from the stools of the probiotic-administered infants via intermediaries healthcare workers. None of the seven in non-probiotic administered rooms was positive.

6. At point B, none of the environmental samples were positive whereas one of 44 stools sample was positive. The latter is intriguing as probiotic administration had been stopped at least three months before and environmental samples were negative. The inference that is deduced from this is that it is from healthcare workers who were still harboring the probiotic bacteria. This buttresses our previous argument that probiotic bacteria bear the characteristics of resident skin flora and are not easily removed by the usual hand disinfection routines of the nursery.

However, absolute safety with probiotic use in preterm neonates has been demonstrated in both these trials and cross contamination was in effect a welcome phenomenon that extended the benefits of probiotics to the control group neonates as well masking the true benefits of probiotic administration [6, 11].

Putative mechanisms of probiotic bacteremia

There have been about five reported cases of bacteremia and systemic sepsis during probiotic therapy (four with lactobacillus and one due to bifidobacterium) and these have been mainly in patients with open intestinal malformations like omphalocele [13]. Therapy with the probiotic yeast Saccharomyces boulardii also results in occasional cases of fungemia especially in those with central venous indwelling catheters [7, 13, 14]. The first author had previously reported his experience with the routine use of Saccharomyces supplementation in neonates with birth weight 1000 to 1999 g from a corporate hospital in South India [13] and has seen a case of putative probiotic fungemia post the study period in a preterm neonate with central vein that responded to fluconazole therapy. But no instances of probiotic bacteremia were seen in the UK and Australian studies on probiotic bacteria use in preterm neonates that included babies less than 1000 g as well with the standard exclusion of babies with major congenital malformation [3,4]. The instances of fungemia that had occurred during treatment with saccharomyces boulardii in patients with central venous catheters are probably due to the fact that preparation of the supplement was done by the bedside. In the UK PiPS study although the proportion of neonates with central venous catheters has not been mentioned (given the intensive care days needed for their care, central venous catheters would have been in place for at least some of them), the preparation of probiotic dose away from bedside besides active infection control policies against catheter-associated blood-stream infections were successful impediments towards systemic bacteremia. We infer from these facts that colonization of the gut with probiotics is safe and confers benefits and iatrogenic systemic infections are probably the result of direct surface contamination of central venous catheters and exposed gut.

## Discussion

It can be logically deduced from this article that cross-contamination (as termed by PiPS Study) or cross-colonization (as termed by ProPrems study group) does exist with probiotic bacteria [3, 4]. Both environmental transmissions (by colonization of environment) as well as transmission by healthcare workers have their respective roles in this phenomenon. The true beneficial effects of probiotic bacteria are masked to a great extent by this phenomenon that confers the advantages of probiotic bacteria administration to the non-study group as well. The significant difference in the cross-colonization rates of the UK and Australian units implies that the infection control and nursery disinfection routines that are of existence currently in different neonatal units and geographical domains vary in their efficacy. As absolute safety with probiotic use has been documented in these larger and rigorous randomized controlled studies from the UK and Australia, further putative therapeutic uses of probiotics can now be explored.

Therapeutic and research implications of the cross-colonization phenomenon of probiotic bacteria

Colonization of the gut with gram-negative enteric pathogens and subsequent translocation has been proposed as the causative factor in a majority of blood-stream infections in neonates [15, 16]. In the Italian study mentioned at reference 15, biweekly rectal swabs were done to evaluate colonization with multidrug-resistant gram-negative bacilli and 55% of sampled neonates were colonized and 72% of these were due to cross-colonization as proven by molecular analysis [17]. Introduction of routine probiotics in such units may confer beneficial effects in prevention of translocation and subsequent blood-stream infections and needs to be investigated further. It can be further postulated that the novel gram-negative probiotic bacteria E. coli Nissle 1917 that has a persistent gut colonization effect can be an effective single dose weapon in our fight against multidrug-resistant gram-negative infections [16].

Efficacy of the standard nursery disinfection routines and hand hygiene standards are not consistent across different centers which is reflected in the lower cross-colonization rate observed in the Australian units who follow the State of Victoria Cleaning Standards for Health facilities [18]. This, when coupled with the sentinel World Health Organization document, the WHO guidelines on hand hygiene in healthcare 2009 [8], would imply that more research is needed to improve our understanding of microbial transmission in hospital care settings and to improve our current disinfection routines and standards.

## Conclusions

Cross colonization phenomenon of probiotic bacteria does exist and by virtue of it even the control group neonates in the well designed large randomized trials of probiotic use in neonates derive the advantages that were thought to accrue to the study group infants only. Both environmental factors and healthcare workers’ hand hygiene are contributory factors in the genesis and existence of this phenomenon. Probiotic bacteria are resilient to eliminate by the currently recommended disinfection routines and hand hygiene routines. It is probable that this phenomenon can be an effective weapon in our fight against multi-drug resistant pathogenic neonatal bacteria.
